# Valve‐shaped thrombus underneath an aortic bioprosthesis

**DOI:** 10.1111/jocs.15845

**Published:** 2021-07-26

**Authors:** Giorgia Cibin, Augusto D'Onofrio, Assunta Fabozzo, Vincenzo Tarzia, Gino Gerosa

**Affiliations:** ^1^ Division of Cardiac Surgery University of Padova Padua Italy

**Keywords:** cardiovascular pathology, perfusion, replacement, valve repair

## Abstract

We describe massive thrombus formation completely occluding an aortic bioprosthesis in a patient with venoarterial extracorporeal membrane oxygenation and apical venting. The thrombus was surgically removed and the patient recovered with no complications. Timely identification and immediate surgical removal of thrombi may allow patient recovery with no severe complications.

A 35‐year‐old man with severe aortic regurgitation, ascending aortic aneurysm, and severe left ventricular dysfunction underwent aortic valve and ascending aorta replacement. Due to difficult weaning from cardiopulmonary bypass caused by biventricular dysfunction, postcardiotomy extracorporeal membrane oxygenation (ECMO) was implanted. Two days later, a transesophageal echocardiogram confirmed the important biventricular dysfunction with a preserved motility of the aortic bioprosthesis cusps with normal trans‐valvular gradients. Due to clinical evidence of pulmonary edema a vent cannula was placed through a transapical access to allow a better unloading of the left ventricle. Furthermore, ECMO flow was increased to provide 75% of cardiac output. After the procedure, *we start intravenous heparin infusion aiming to maintain activated partial thromboplastin time (aPTT) between 40 and 50 s and we monitor blood coagulation every 6 h. In this case, a spontaneous anticoagulation pattern was identified (plts: 73 × 10^9^/L; spontaneous INR: 2.12; PT: 34%; aPTT: 40 s), therefore heparin infusion was started at low dosage (6250 IU/day)*. A few hours later, ECMO flow suddenly reduced resulting in severe hemodynamic impairment. A transesophageal echocardiography showed massive thrombosis of the aortic bioprosthesis and of the ascending aorta requiring emergent surgical revision. During surgical re‐exploration, thrombi from the ascending aorta and from the aortic side of the bioprosthesis were removed (Video S1). Furthermore, a valve‐shaped thrombus (Figure 1A) molded on the ventricular side of the aortic bioprosthesis and completely occluding the left ventricular outflow tract, was found and removed (Figure 1B). Thrombus formation was due to the absence of blood flow through the aortic valve that remained closed due to the left ventricular venting, despite the spontaneous anticoagulation pattern and heparin infusion. The patient had no neurologic injury and underwent left ventricular assist device implantation but died few months later for cerebral hemorrhage.

**Figure 1:**
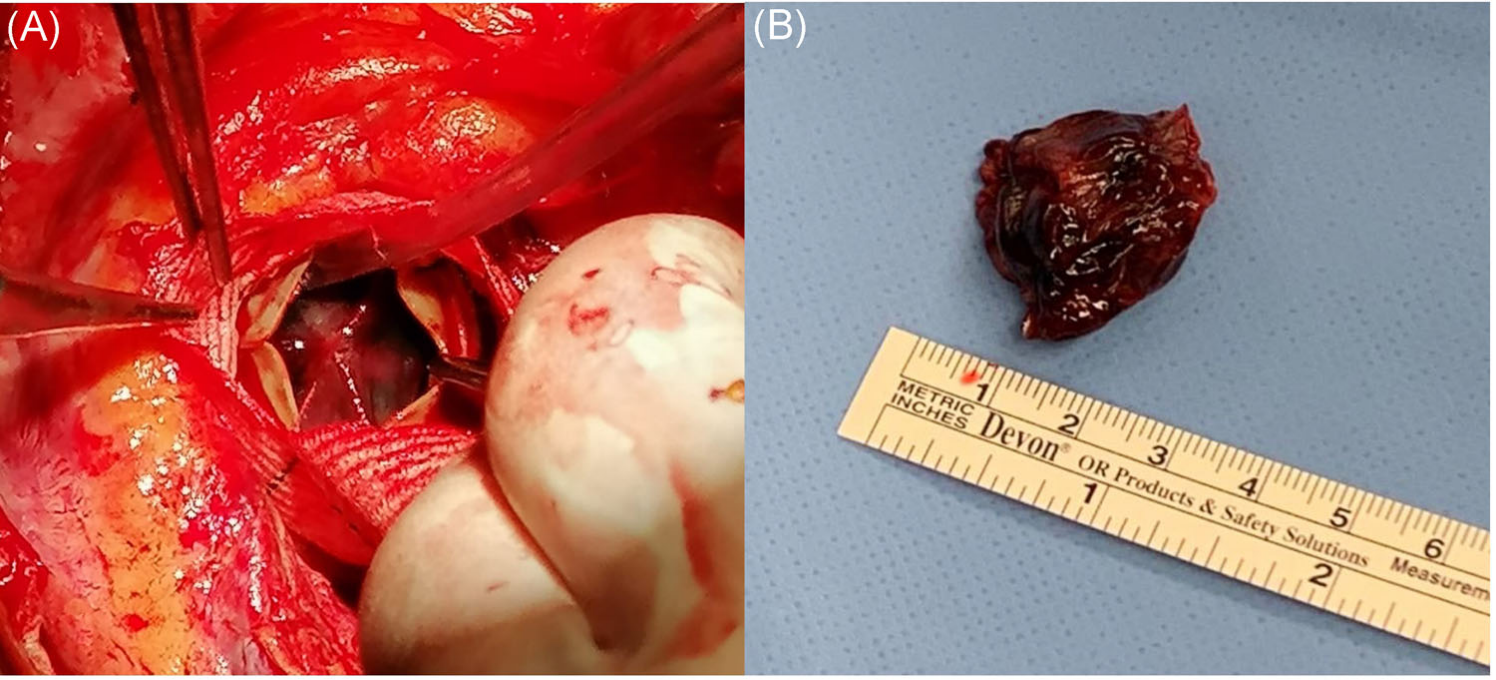
Intraoperative image showing the valve‐shaped thrombus, completely occluding the left ventricular outflow tract, visible through the open leaflets of the aortic bioprosthesis (A) and after removal (B)

*Thrombus formation in patients supported by ECMO has been already described and it has been shown to be associated with high mortality. A recent paper by Williams and Bernstein* ^1^ *describes 12 patients who developed intracardiac thrombosis with only two survivors*. *Therefore, prevention of thrombus formation is of utmost importance in such a delicate population*. This case is significant for several reasons: (1) ECMO configuration with left ventricular venting decreases, and often abolishes completely blood flow through the aortic valve thus seriously increasing the risk of valve thrombosis; (2) although heparin infusion and demonstrated anticoagulation at blood exams, valve thrombosis may occur with this ECMO configuration and strict echocardiographic monitoring is mandatory; (3) timely identification and immediate surgical removal of thrombi may allow patient recovery with no severe complications. *An alternative strategy to vent the left ventricle during ECMO support is represented by the Impella system in the so‐called ECMELLA configuration*.^2^, ^3^ *This has the advantage of reducing the risk of thrombus formation due to excessive drainage and consequent aortic valve closure; on the other hand it is an expensive device and it is not available in all centers*.

## CONFLICT OF INTERESTS

The authors declare that there are no conflict of interests.

## ETHICS STATEMENT

Written informed consent was obtained for publication.

## Supporting information

Supporting information.
